# Treatment of Aggressive Prolactin-Secreting Pituitary Adenomas with Adjuvant Temozolomide Chemotherapy: A Review

**DOI:** 10.7759/cureus.658

**Published:** 2016-06-27

**Authors:** Marc Moisi, Aurora S Cruz, Tara Benkers, Steven Rostad, Frances Broyles Broyles, Kevin Yuen, Marc Mayberg

**Affiliations:** 1 Seattle Science Foundation; 2 Neurological Surgery, Wayne State University; 3 Neurological Surgery, University of Louisville; 4 Neurological Surgery, University of California, Irvine; 5 Neurological Surgery, Swedish Neuroscience Institute; 6 Neuro-oncology, Swedish Neuroscience Institute; 7 Pathology, CellNetix; 8 Endocrinology, Swedish Neuroscience Institute; 9 Swedish Pituitary Center, Swedish Neuroscience Institute; 10 Swedish Neuroscience Institute

**Keywords:** aggressive pituitary tumor, prolactinoma, radiosurgery, temozolomide

## Abstract

Most prolactin-secreting pituitary adenomas demonstrate slow growth and are effectively managed with medical/surgical therapy. Rarely, these tumors can behave aggressively with rapid growth and invasion of local tissues, and are refractory to medical, surgical, or radio-surgical therapies. We report a case of a prolactin-secreting adenoma in a young woman, which became progressively aggressive and refractory to usual treatment modalities, but responded to treatment with the chemotherapeutic agent temozolomide. In addition, we review the literature for treatment of refractory adenomas with temozolomide. The clinical and pathologic characteristics of aggressive prolactin-secreting adenomas are reviewed, as well as their response to dopamine agonists, surgery, radiotherapy, and chemotherapy.

## Introduction and background

Pituitary adenomas are one of the more common intracranial neoplasms [1]. They tend to grow slowly without invasion and usually respond to medical therapy with dopamine agonists. However, these tumors rarely may exhibit aggressive behaviors invading local structures with multiple recurrences that do not respond to standard treatments of medical therapy, resection, and adjuvant radiation [2]. We describe a case of a patient with a prolactin-secreting adenoma with multiple recurrences over 10 years, which became progressively invasive and resistant to standard therapies, including dopamine agonists and trans-sphenoidal surgery, but responded well to adjuvant chemotherapy with temozolomide (TMZ).

### Illustrative case

**Figure 1:**
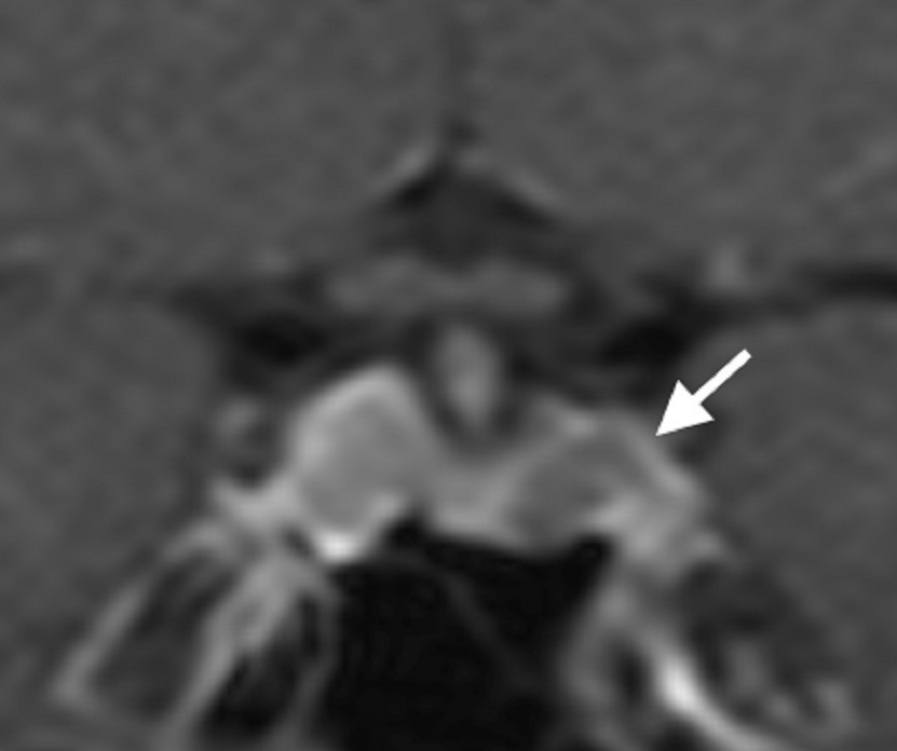
Coronal Post-Contrast MRI Sequences Following initial successful treatment with bromocriptine (arrow shows tumor within gland)

A 37-year-old gravida-1, para-0 female presented in 2002 with secondary amenorrhea and galactorrhea. She described the onset of menses at age 13 and had normal menstrual periods through her teens. She was on birth control pills until 1999 with normal menstrual periods. After stopping birth control pills, her periods did not resume, and she noted intermittent galactorrhea. Evaluation revealed an elevated prolactin level of 110 ng/ml (normal <24 ng/ml), and MRI demonstrated a 10 mm pituitary lesion. She also had central hypothyroidism and was started on levothyroxine. She was placed on bromocriptine and had an excellent initial response with normalization of her prolactin and resumption of menstrual periods. Follow-up MRI showed regression of the tumor to 7 mm shown as in Figure 1.

**Figure 2:**
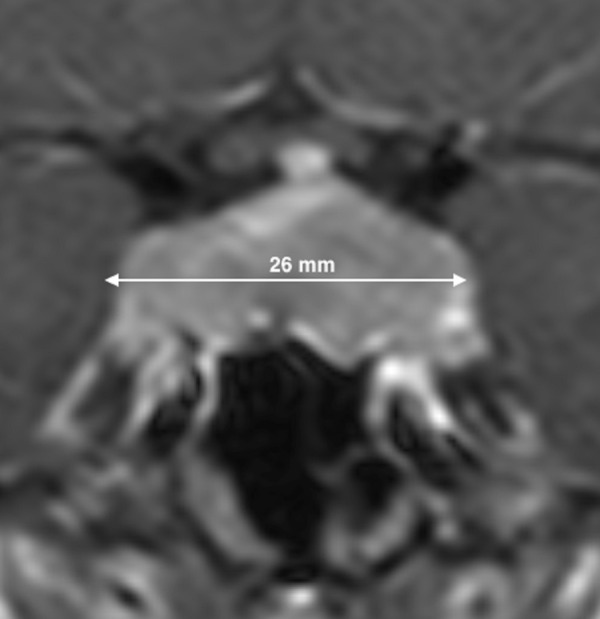
Coronal Post-Contrast MRI Sequences First recurrence despite maximal medical therapy, showing marked enlargement and invasion of both cavernous sinuses

After six months, however, amenorrhea recurred with an elevation of prolactin to 55 ng/ml despite maximal tolerable bromocriptine therapy (15 mg daily), and in Figure 2 is an MRI that showed an increase in the tumor size to 26 mm in diameter with suprasellar extension and probable invasion to both cavernous sinuses.

**Figure 3:**
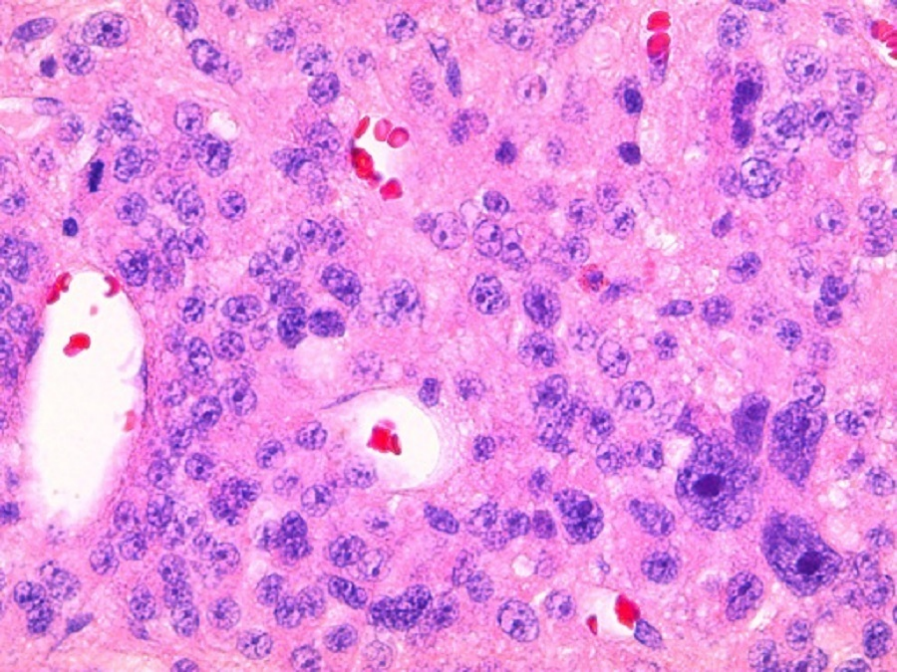
Photomicrographs of Histologic Specimens from First Surgery (400x) Hematoxylin and eosin stain demonstrating pituitary adenoma with increased nuclear pleomorphism and elevated mitotic activity

**Figure 4:**
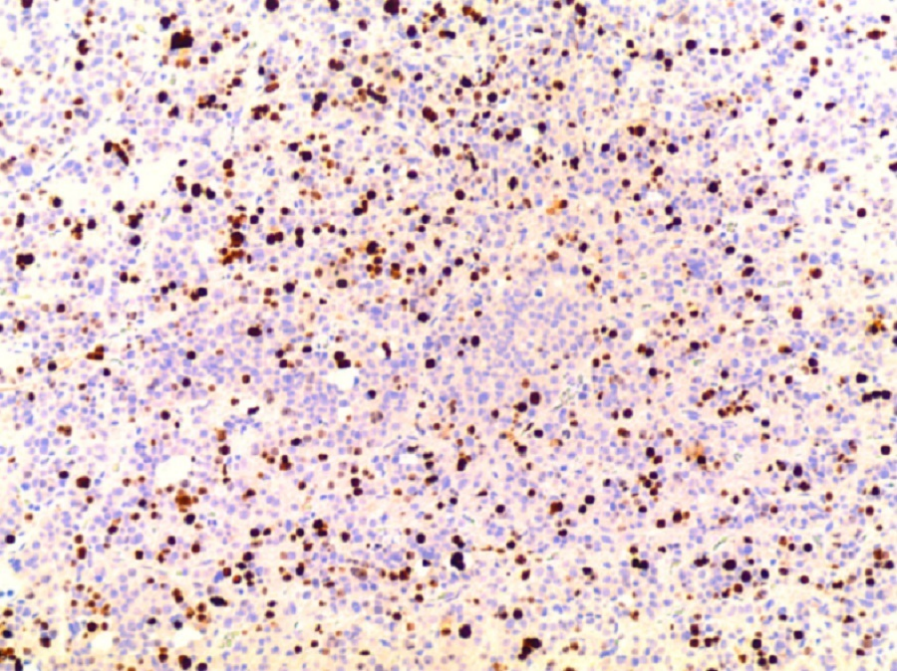
Photomicrographs of Histologic Specimens from First Surgery (100x) Increased immunoreactive Ki-67 labeling index

A trans-sphenoidal adenomectomy was performed in June 2005, with subsequent normalization of prolactin levels (11.3 ng/ml). Pathology was consistent with a pituitary adenoma showing sparse immunoreactive prolactin, with increased nuclear pleomorphism, elevated mitotic activity, and increased p53 labeling suggesting increased biologic aggressiveness and invasion as shown in Figure 3 and Figure 4.

**Figure 5:**
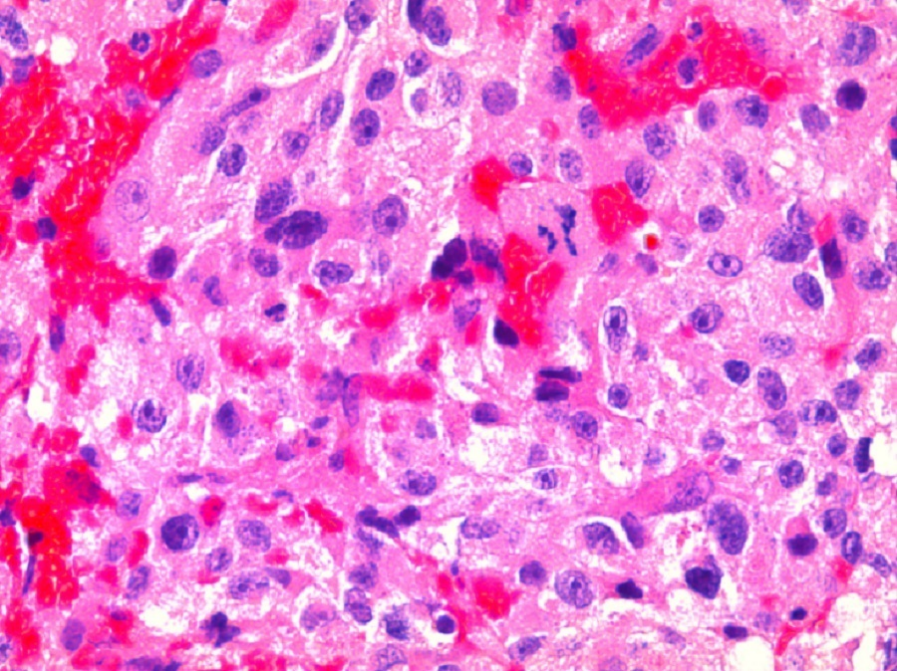
Photomicrographs of Histologic Specimens from Final Surgery Hematoxylin and eosin stain with additional nuclear pleomorphism and mitoses

**Figure 6:**
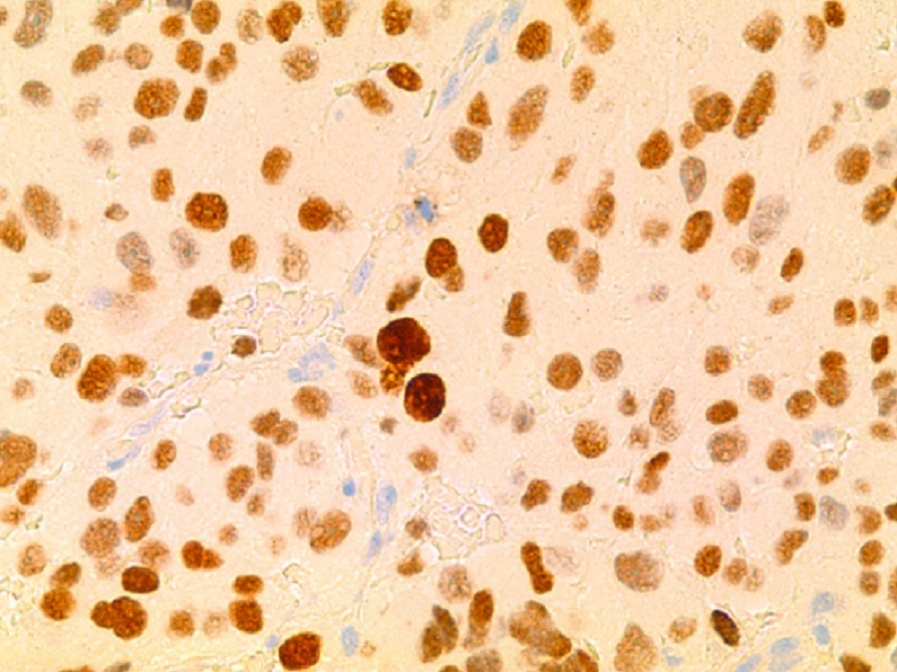
Photomicrographs of Histologic Specimens from Final Surgery (400x) Markedly increased p53 labeling

**Figure 7:**
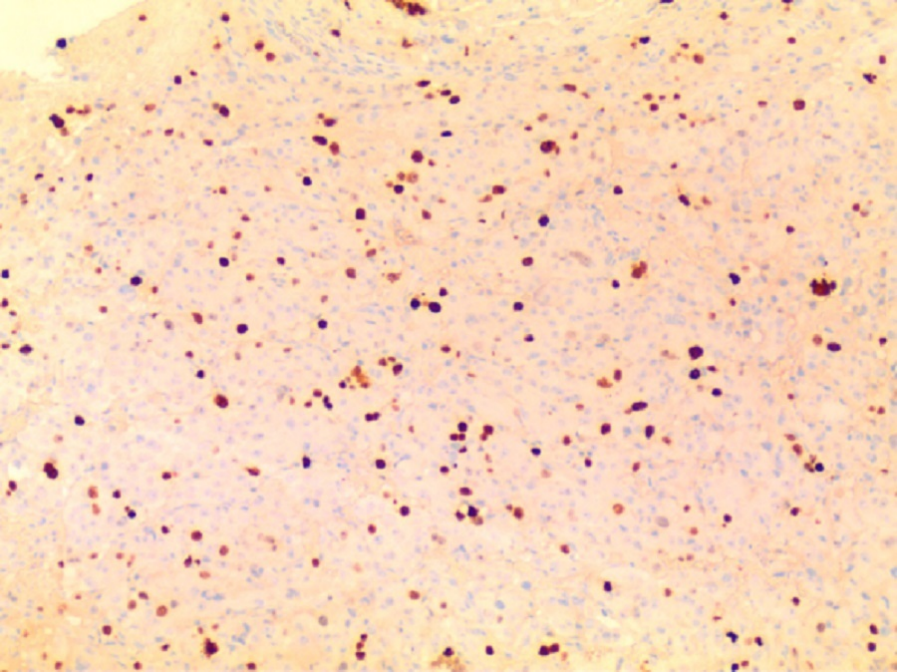
Photomicrographs of Histologic Specimens from Final Surgery (100x) Markedly increased Ki-67 labeling index

Due to these findings on pathology, further treatment with stereotactic radiosurgery was recommended, but the patient declined due to concerns regarding infertility. Over the ensuing year, the tumor continued to increase in size to 17 mm diameter with further invasion of the cavernous sinuses. She subsequently developed headaches as well as right third and sixth nerve palsies, which resolved following a second trans-sphenoidal debulking surgery in June 2006. In November 2006, she underwent single-dose Cyberknife radiosurgery (marginal dose 22 Gy). However, she became amenorrheic despite normalization of her prolactin level and lack of radiographic progression for four years. Repeat MRI in 2010 showed further progression of the tumor into the right cavernous sinus, for which she was treated with Gamma Knife radiosurgery in January 2011 (20 Gy single fraction). The clinical status and MRI studies remained stable for the next three years, but in 2014, she developed progressive right third and sixth nerve palsies and MRI showed further tumor growth up to 3.4 x 3.1 x 3.8 cm. Because she was not a candidate for further radiosurgery, she underwent another trans-sphenoidal debulking surgery, and at that time, the decision was made to commence TMZ chemotherapy in October 2014. Pathology was consistent with an atypical prolactinoma with increased nuclear pleomorphism, marked elevation of the Ki-67 labeling index to 25%, and p53 labeling index to 70%. These histologic findings are shown in Figures 5-7 below.

**Figure 8:**
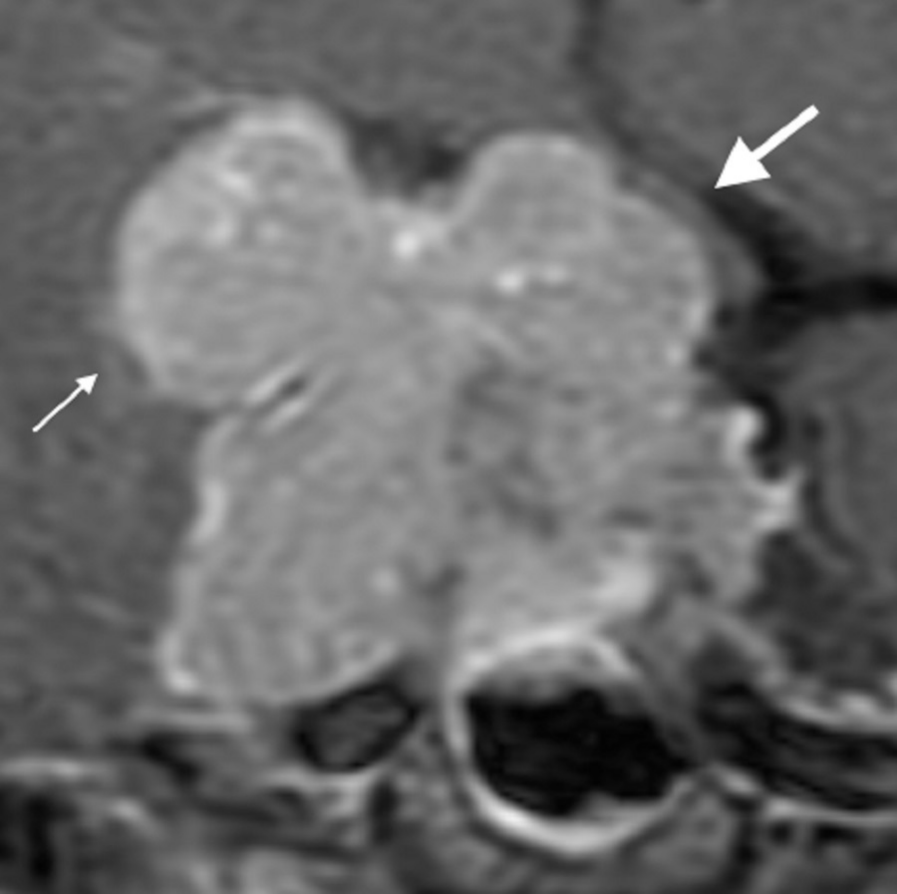
Coronal Post-Contrast MRI Sequences Prior to craniotomy, demonstrating marked expansion with suprasellar extension and compression of optic chiasm (arrow) and intracranial invasion into right middle fossa

Next Generation sequencing of her tumor identified mutations in TP53, IDH2, CDH1, and KIT. O(6)-methylguanine methyltransferase (MGMT) proliferation markers were not done on this particular case. While awaiting insurance authorization for TMZ therapy, she developed visual field deficits in her left eye as well as radiographic progression of her tumor as shown in Figure 8.

**Figure 9:**
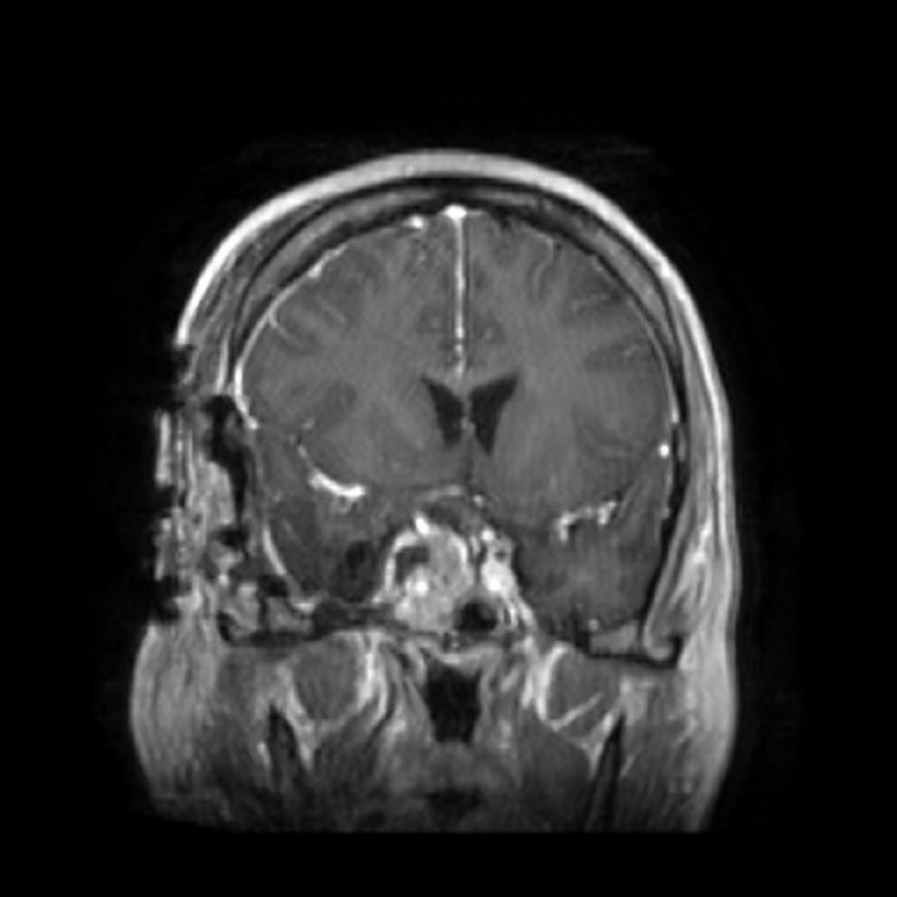
Coronal Post-Contrast MRI Sequences After craniotomy at initiation of TMZ treatment, showing decompression of optic chiasm and debulking of tumor

She then underwent a right, frontotemporal craniotomy in January 2015 for decompression of the optic nerves and chiasm, with postoperative restoration of full visual fields. The postoperative MRI is shown in Figure 9.

**Figure 10:**
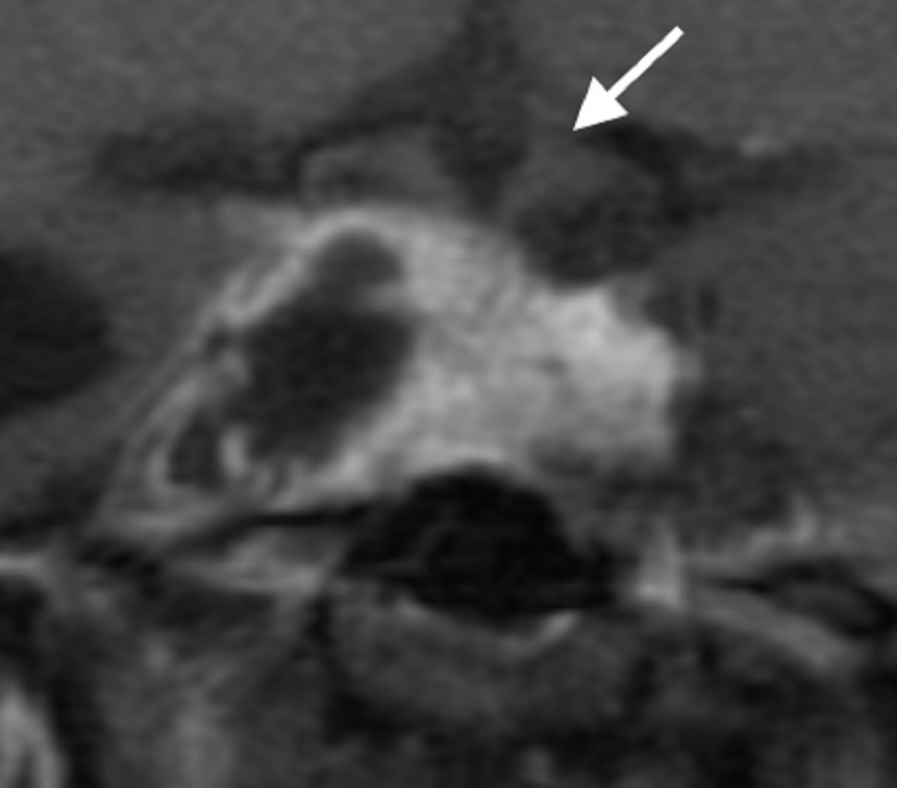
Coronal Post-Contrast MRI Sequences After Cyberknife radiosurgery

The histology was similar to the 2014 pathology. Given the rapid recurrence and intracranial invasion, she was treated with fractionated Cyberknife radiosurgery over 5 fractions (25 Gy), followed by a course of chemotherapy with 12 cycles of TMZ given on a five-day schedule every 28 days at 150 mg/m^2^ for the first cycle and increased to 200 mg/m^2^ for subsequent cycles. MRI of the brain two months following completion of radiation, in Figure 10, showed stable tumor compared to the postoperative MRI.

**Figure 11:**
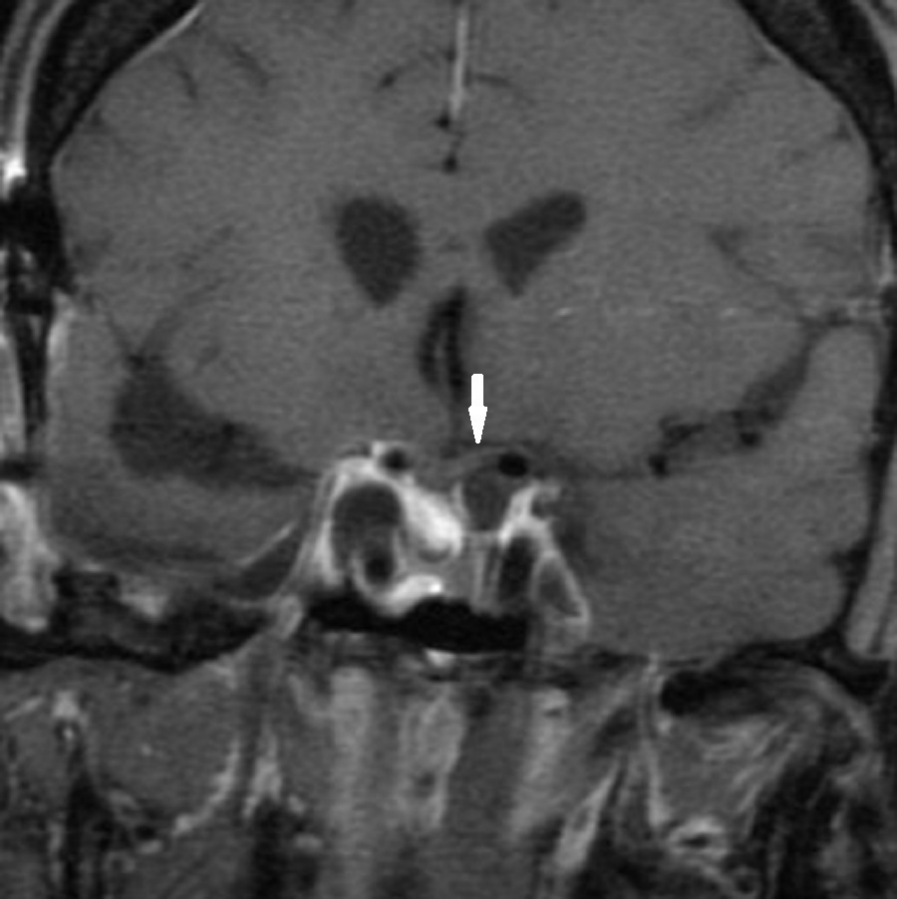
Coronal Post-Contrast MRI Sequences Sequential tumor regression over eight cycles of TMZ treatment. Note the prominent reduction in tumor volume and gadolinium enhancement.

**Figure 12:**
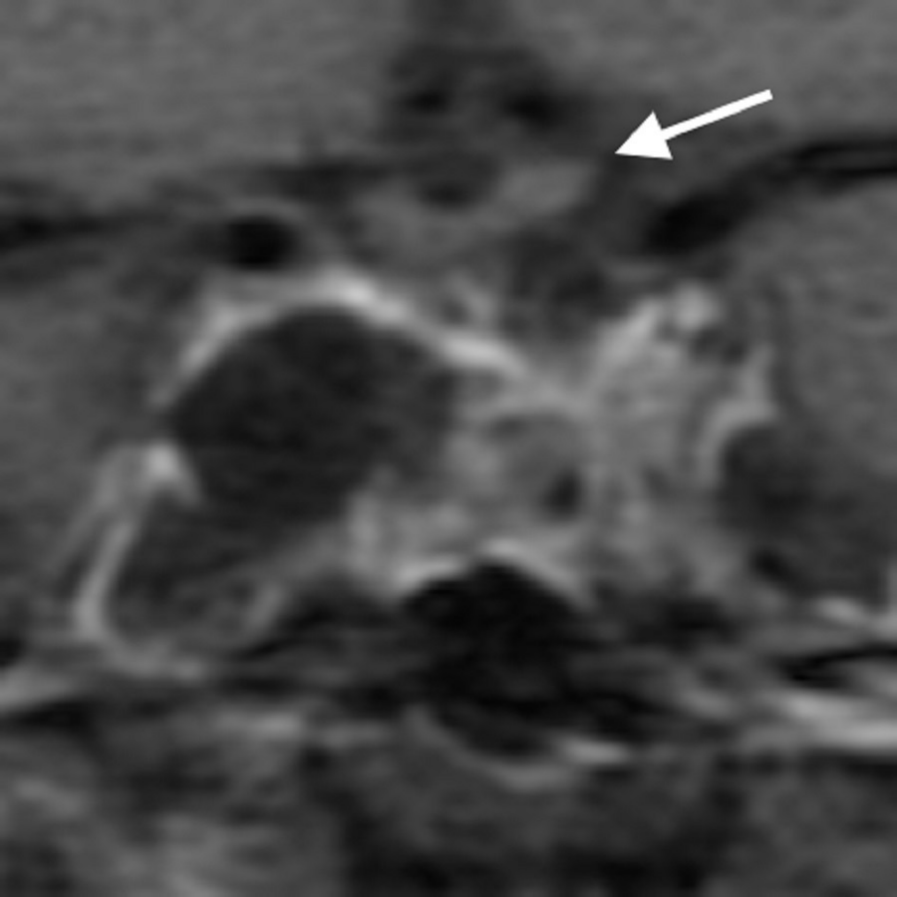
Coronal Post-Contrast MRI Sequences Sequential tumor regression over eight cycles of TMZ treatment. Note the prominent reduction in tumor volume and gadolinium enhancement.

Following eight cycles of TMZ to date, surveillance MRI scans--demonstrated in Figures 11-12--show continued decrease in the enhancing sellar and suprasellar tumor, with continued clinical improvement in the patient’s right third palsy, consistent with a good response to chemotherapy.

## Review

Prolactinomas, which represent 40% of all pituitary tumors, are the most common variant, with an incidence of 27 cases per million persons per year and a higher incidence in females of child-bearing age [1-2].  Lactotrophs within the tumor are inhibited by endogenous dopamine or exogenous dopamine agonists [2]. This release of prolactin stimulates milk production, inhibits gonadotropin-releasing hormone secretion, and directly impairs gonadal steroidogenesis, thereby causing the typical symptoms of hypogonadism, amenorrhea, and galactorrhea [1-2]. Prolactinomas are typically benign and remain stable or slowly increase in size, rarely causing mass effect symptoms [2]. When mass effect is present, visual symptoms related to optic chiasm compression may occur in addition to possible hypopituitarism or stalk compression [2]. As the majority of prolactinomas are benign microadenomas (<1cm), primary treatment is aimed at controlling the secretion of prolactin using dopamine agonists such as cabergoline or bromocriptine [2]. These pharmacologic approaches are effective, reducing serum prolactin levels by nearly 50% in nearly all patients and the size of the tumor by greater than 25% in the vast majority of patients [2]. In prolactinomas with mass effect or resistance to dopamine agonist medical therapy, surgery/radiosurgery may be indicated, though these therapies may be associated with a higher rate of inducing a worsening of hypopituitarism and higher recurrence rates [2].

*Refractory prolactinoma* is defined as a tumor in which dopamine agonist therapy is ineffective and where surgical resection is unsuccessful [3]. In addition to resistance to medical therapy, these tumors often demonstrate early recurrence and rapid local growth after surgery/radiotherapy [4]. *Aggressive prolactinomas* likely represent a further subset of refractory prolactinomas. Fortunately, aggressive pituitary adenomas are rare, comprising between 2.7%-15% of all pituitary adenomas [3]. There is much debate and discussion regarding what constitutes an 'aggressive' adenoma and invasion [2-3]. Though there are no clear histologic features to confirm the diagnosis of an aggressive adenoma, the 2004 WHO pituitary tumor classification system defined 'atypical adenomas' that are distinct from benign adenomas but without distant or craniospinal metastatic disease, as observed with pituitary carcinomas. This histologic 'atypical adenoma' category attempts to assess aggressiveness of adenomas based on morphologic and growth-related features, including high mitotic index, increased immunohistochemical p53 staining, and an elevated Ki67 labeling index >3% [4]. The abnormal p53 staining most likely correlates with the TP53 mutation of the patient’s genetic sequencing data. Both findings are rare in adenomas and are associated with invasiveness and aggressive growth [5-6]. Although this patient’s tumor showed pathologic and clinical features corresponding with atypical adenoma, this variant is not consistent in identifying clinically aggressive adenomas, and debate remains regarding the definition and true incidence of these tumors due to the lack of consensus on diagnostic criteria [2-3]. Morphologic (hormonal) classification remains the most reliable method to predict behavior. Different pathologic criteria for atypia may need to be developed to reflect these unique tumor types.

In a retrospective study of 410 patients where characteristics that were predictive of aggressive behavior were defined, Trouillas, et al., proposed a classification system based on combining histochemical proliferative markers (two or more of the following: Ki67 index ≥3%; >2 mitoses per 10 HPFs; or p53 immunopositivity) and radiographic evidence of cavernous or sphenoid sinus invasion [7]. Using the Trouillas criteria, patients with Grade 2b (invasive and proliferative) adenomas had a higher probability of persistence or recurrence of 12-fold and 25-fold, respectively [7]. This study also found that radiographic evidence of cavernous or sphenoid sinus invasion alone was superior to histologic markers when assessing the degree of aggressiveness of these adenomas [7]. In our case, despite only modest elevation levels in prolactin, the tumor showed progressively more aggressive characteristics, including cavernous sinus and intracranial invasion, histologic evidence of increased mitosis and markedly elevated proliferation index, and p53 positivity. In this regard, the tumor recurred multiple times despite medical therapy, multiple surgical resections, and radiosurgery.

Although currently there is not a single, reliable immunohistochemical marker, imaging characteristic or universally accepted definition for aggressive adenomas; it is believed that benign adenomas may undergo genetic transformation, which may lead to invasive behavior and may eventually transform into malignant pituitary carcinomas [4, 7-8]. In our case, the tumor initially responded to medical therapy and subsequent debulking, with extended periods of clinical quiescence. However, after several years, the tumor became highly invasive and showed clinical and radiographic growth over a matter of weeks. Although there has been rapid accumulation of data on the molecular and genetic findings in pituitary adenomas, no common mutational events have been found to explain tumorigenesis [9]. Furthermore, studies have identified few markers that predict invasiveness or proliferation. However, there are retrospective studies that have demonstrated transformation of previously benign tumors into pituitary carcinoma after recurrence [4].

The challenge of treating an aggressive adenoma is inherent in its diagnosis – these tumors are resistant to medical, surgical, and radiotherapy treatments. When approaching benign micro and macroprolactinomas, initial treatment is a dopamine agonist such as cabergoline or bromocriptine, both of which have been shown to reduce tumor size, normalize serum prolactin levels, and restore gonadal function in more than 70-95% of patients [2, 5, 8]. If the tumor increases in size or hyperprolactinemia does not resolve with pharmacologic treatment, transsphenoidal surgical resection should be considered [2, 8]. As previously discussed, pharmacologically or surgically resistant tumors, especially with cavernous or sphenoid sinus invasion, should be monitored closely for recurrence [2, 7-8].

When clinical and radiographic evidence leads to the diagnosis of an aggressive adenoma, repeated surgical debulking can be helpful, though these treatments are palliative and often will lead to surgical complications including visual field deficits, infection, cerebrospinal fluid leaks, and panhypopituitarism [4]. Medically resistant and potentially aggressive prolactinomas are more commonly seen with macroprolactinomas (>1 cm), which, in one retrospective study were found to have a disappointingly low prolactin normalization rate of 36% after surgical resection, as opposed to prolactin normalization in 75% of medically refractory microprolactinomas [5].

Radiotherapy is often used in conjunction with surgery to treat aggressive or recurrent pituitary adenomas. Radiotherapy aims to slow or stop tumor growth, restore normal prolactin levels, and minimize radiation dose toxicity [6]. Radiotherapy modalities include external beam radiation therapy (EBRT) and stereotactic radiosurgery (SRS). Both have been shown to be approximately equivalent in normalizing hyperprolactinemia (34.1% for EBRT vs. 31.4% for SRS) [6]. Unfortunately, prolactinomas are the least radiosensitive pituitary tumors, with one study revealing only an 18% remission rate at four years for prolactinomas treated with Gamma Knife radiosurgery [5]. Interestingly, Cohen-Inbar et al., found that Gamma Knife radiosurgery successfully normalized prolactin levels in 50% of patients with dopamine-agonist resistant prolactinomas, although cavernous sinus invasion was a significant predictor of failure to achieve normal prolactin levels [10].

TMZ, an oral alkylating chemotherapeutic drug used primarily for the treatment of brain tumors such as glioblastoma multiforme, has been used for the past 10 years for pituitary carcinomas and aggressive pituitary adenomas [11]. TMZ primarily acts by methylating DNA bases, thereby inducing DNA fragmentation by base mismatch of the repair enzymes. TMZ was reported by Syro et al., in 2006 to successfully treat an aggressive prolactinoma and several cases have been described in the literature since [9, 12]. It is believed that expression of the DNA repair enzyme O^6^-methylguanine-DNA methyltransferase (MGMT) by tumor cells can predict clinical response to TMZ therapy, with multiple studies demonstrating a strong inverse relationship between low MGMT expression and high responsiveness to TMZ and conversely, high MGMT expression with resistance to TMZ [3, 12-13]. TMZ is typically dosed at 150–200 mg/m^2^ for five days in 28-day cycles [11-12]. Several studies have demonstrated that lack of response after three cycles, either in tumor size or prolactin levels, is predictive of a poor outcome [12]. The literature contains prior reports of aggressive pituitary adenomas successfully treated with TMZ, most with rapid shrinkage of the tumor and reduction of serum prolactin levels demonstrated in Table 1 [13-26]. Aggressive prolactinomas appear to have the highest response rate to TMZ at 73%, followed by ACTH-secreting tumors and non-functioning adenomas [13].

**Table 1:** Literature Review of Aggressive Pituitary Adenomas Successfully Treated with TMZ PRL= prolactin RT = radiotherapy TMZ = temazolamide

Author [reference]	Year	Number of Patients with PRL Secreting Adenomas	Treatment Modality	Follow-up	Outcome
Bengtsson D, et al. [27]	2015	11	TMZ + resection, 8 with RT	4-91 months	7/11 responded to TMX therapy. 4/11 with progressive growth despite TMX therapy.
Bruno, et al. [10]	2015	1	TMZ + resection + RT	1 month	Deceased within one month of starting TMZ therapy
Bush, et al. [14]	2010	1	TMZ + resection + RT	unknown	80% reduction in tumor volume, clinical improvement
Byrne, et al. [15]	2009	1	TMZ + resection + RT	6 months	Radiographic and clinical response with stable size and prolactin levels
Fadul, et al. [16]	2006	1	TMZ + resection + RT	15 months	Radiographic and clinical response remaining stable at 15 month follow-up
Hagen, et al. [17]	2009	2	TMZ + resection	12-34 months	Radiographic and clinical response with no increase in tumor size after cessation of TMZ treatment
Kovacs, et al. [18]	2006	1	TMZ + resection + RT	10 months	Radiographic and clinical response
Lim, et al. [19]	2006	1	TMZ + resection + RT	24 months	Radiographic and clinical response with stable size and prolactin levels
Losa, et al. [20]	2010	2	TMZ + resection + RT	12-21 months	1 with radiographic and clinical response and tumor control at last followup at 12 months, 1 with stable tumor size and no tumor control at last follwup at 21 months. Both patients living at last follow-up.
McCormack, et al. [21]	2009	1	TMZ + resection + RT	4 months	1 patient with good radiographic and clinical response
Murakami, et al. [22]	2011	1	TMZ + resection + RT	11 months	Initial radiographic and clinical response with subsequent tumor growth and death
Neff, et al. [24]	2007	1	TMZ + resection + RT	26 months	Radiographic and clinical response with stable size and prolactin levels on chronic TMZ therapy
Whitelaw, et al. [3]	2012	3	TMZ + resection	18-24 months	Radiographic and clinical response
Zemmoura, et al. [26]	2012	1	TMZ + resection + RT	unknown	Failure to respond

In our case, the patient had an aggressive prolactinoma resistant to dopamine agonist pharmacotherapy, with recurrence after multiple surgical resections and radiotherapy. TMZ was started as a salvage therapy for recurrent mass effect and neurological deficits in conjunction with radiosurgery. After eight cycles of TMZ, the patient responded with excellent clinical results on her MRIs demonstrating continued radiographic regression of her tumor coincident with clinical improvement. The excellent response rate of progressive pituitary adenomas treated with TMZ therapy, combined with the relatively low morbidity of this drug, raises the question as to whether TMZ should be considered as an initial component of treatment at the time tumor recurrence is detected. 

## Conclusions

TMZ for the treatment of aggressive pituitary adenomas, especially prolactinomas, has shown promise in controlling these invasive and highly aggressive tumors. Based upon reported anecdotal experience, TMZ may be more effective than radiotherapy as a second-line treatment after surgery. Although this patient’s tumor is an example of atypical adenoma histology and immunohistochemistry, more research is needed to better define and discover accurate biomarkers for aggressive pituitary adenomas for earlier diagnosis and prediction of treatment response. Future prospective clinical trials should be considered to evaluate the role of TMZ in the management of patients with aggressive pituitary adenomas. The relationship between MGMT and TMZ sensitivity also requires further understanding and may lead to better patient selection and tailored treatments for these invasive tumors. 
